# Supplementary material for: Managing low-back pain in rural Uganda: a qualitative study exploring the perspectives and practices of frontline health workers regarding LBP management in primary care
Source: BMC Musculoskelet Disord. 2025 Feb 19;26:168. doi: 10.1186/s12891-024-08164-9 (PMC11837481; doi:10.1186/s12891-024-08164-9)
Supplement: Supplementary file 1 — Supplementary Material 1 [file 12891_2024_8164_MOESM1_ESM.pdf]

Participant n	Gender	Age	Occupation	HC3 or 4?	Level of training	
16	M	50	CO	4	Dip Clin Med and Com Health Dipl in health Admin	COS
7	F	32	CO	3	Diploma in clinical medicine	
8	F	34	Nurse	3	Nursing	
17	F	35	Nurse	4	Dip Gen Nursing	
19	F	32	Nurse	3	Nursing	
18	F	36	Nurse	3	Nursing	
11	F	31	CO	3	Dip Clin Med and Com Health	GNC
10	F	50	Nurse	4	Dip Nursing	
4	F	30	Nurse	4	Dip Nursing	
6	F	37	CO	3	Dip Clin Med and Com Health	
5	M	29	Nurse	3	Dip Nursing	
2	M	29	Nurse	4	Dip Nursing	
12	F	34	Nurse	3	Dip Nursing	
3	M	34	CO	4	Dip Clin Med and Com Health	PH
15	F	30	Nurse	3	Dip Nursing	
9	M	46	CO	4	Dip Clin Med and Com Health	
1	F	38	CO	4	Dip Clin Med and Com Health	
13	M	35	Nurse	3	Dip Nursing	
14	F	34	CO	3	Dip Clin Med and Com Health	
Participant	Gender	Age	Occupation	HC3 or 4?	Level of training	
number		35.578				
		6.4058				
