# Supplementary material for: Managing low-back pain in rural Uganda: a qualitative study exploring the perspectives and practices of frontline health workers regarding LBP management in primary care
Source: BMC Musculoskelet Disord. 2025 Feb 19;26:168. doi: 10.1186/s12891-024-08164-9 (PMC11837481; doi:10.1186/s12891-024-08164-9)
Supplement: Supplementary file 2 — Supplementary Material 2 [file 12891_2024_8164_MOESM2_ESM.pdf]

Initial questionnaire for healthcare professionals

CHECKLIST FOR INTERVIEWER

- ☐ **CONSENT FORM SIGNED**
- ☐ **INFORMATION LEAFLET PROVIDED**
- ☐ **Demographics questionnaire filled**

https://docs.google.com/document/d/1QWhLAht0sxH-6VvYgbKgbezXJa6szj-V56iOFyrIxBg/edit

Associated sheet: https://docs.goohttps://docs.google.com/document/d/1QWhLAht0sxH-6VvYgbKgbezXJa6szj-V56iOFyrIxBg/editgle.com/spreadsheets/d/1M5E98Jl5nPEJlxjT18j51Z0zqbryGO6KzedTvjc57eU/edit#gid=0

TIME AND LOCATION OF THE INTERVIEW:..

1st Part: Understanding the challenge of LBP.

To what extent is low-back pain a burden in your everyday practice? (*Prevalence, struggle to manage, associated pathologies*)

How do you usually manage low-back pain? (*in terms of medication, complementary exam, etc*)

What are, in your opinion, the reasons why people have LBP?

What are the beliefs associated with LBP?

What are the expectations from people with LBP when they come to you?

2nd Part: Exploring the opinions and perceptions of new technologies in care.

Do you usually use technologies as part of your work? If so, what are they?

Do you feel confident using technology in your work?

What is the purpose of the technology you are using today as part of your work?

3rd Part: The digital clinical decision support system (DCDSS)- BACKTRACK

How would you feel about using BACKTRACK for LBP management in your work? (*If needed, the notion of a digital clinical decision support system can be defined*).

Do you think the questions in BACKTRACK should be translated in your local language? Do you think it should be translated into other languages?

What are your initial impressions of BACKTRACK? Do you have any suggestions?

4th Part: **Education and training needs**

Do you have other training needs with respect to LBP?
